# Selection among critically endangered landlocked salmon (*Salmo salar* m. *sebago*) families in survival and growth traits across early life stages and in different environments

**DOI:** 10.1111/eva.13692

**Published:** 2024-04-26

**Authors:** Matti Janhunen, Aslak Eronen, Jukka Kekäläinen, Craig R. Primmer, Iikki Donner, Pekka Hyvärinen, Hannu Huuskonen, Raine Kortet

**Affiliations:** ^1^ Natural Resources Institute Finland (Luke) Joensuu Finland; ^2^ Department of Environmental and Biological Sciences University of Eastern Finland Joensuu Finland; ^3^ Faculty of Biological and Environmental Sciences|Institute of Biotechnology University of Helsinki Helsinki Finland; ^4^ Natural Resources Institute Finland (Luke) Paltamo Finland

**Keywords:** captive rearing, growth, hatchery selection, salmonids, survival

## Abstract

Endangered wild fish populations are commonly supported by hatchery propagation. However, hatchery‐reared fish experience very different selective pressures compared to their wild counterparts, potentially causing genotype‐by‐environment interactions (G × E) in essential fitness traits. We experimentally studied early selection in a critically endangered landlocked Atlantic salmon population, first from fertilization to the swim‐up stage in a common hatchery setting, and thereafter until the age of 5 months in two contrasting rearing environments. Swim‐up progeny were moved either to standard indoor hatchery tanks involving conventional husbandry or to seminatural outdoor channels providing only natural food. After the first summer, sampled survivors were assigned to their families by genotyping. Early survival until the swim‐up stage was mostly determined by maternal effects, but also involved significant variation due to sires and full‐sib families (potential genetic effects). High on‐growing survival in hatchery tanks (88.7%) maintained a more even distribution among families (relative share 1.5%–4.2%) than the seminatural environment (0.0%–5.4%). This heterogeneity was mostly maternal, whereas no independent paternal effect occurred. Heritability estimates were high for body size traits in both environments (0.62–0.69). Genetic correlations between the environments were significantly positive for body size traits (0.67–0.69), and high body condition in hatchery was also genetically linked to rapid growth in the seminatural environment (0.54). Additive and phenotypic growth variation increased in the seminatural environment, but scaling effects probably played a less significant role for G × E, compared to re‐ranking of genotypes. Our results suggest that not only maternal effects, but also genetic effects, direct selection according to the environmental conditions experienced. Consistently high genetic variation in growth implies that, despite its low overall genetic diversity and long history in captive rearing (>50 years), this landlocked Atlantic salmon population still possesses adaptive potential for response to change from hatchery rearing back to more natural conditions.

## 1 INTRODUCTION

Hatchery propagation is a routine management practice used to support endangered wild fish populations or re‐establish those already extirpated (Fisch et al., 2015; Naish et al., 2007). Owing to stable rearing conditions and unlimited food, hatchery fish experience very different selective pressures compared to their wild counterparts (Frankham, 2008; Waples, 1999). The physiological and behavioral traits needed to thrive in a crowded rearing environment with no predators and abundance of energy‐rich food differ profoundly from the traits that are adaptive in most natural waters (Blouin et al., 2021; Glover et al., 2001, 2004; Saikkonen et al., 2011). Cumulative data on salmonids suggest that developmentally plastic and genetic adaptation to captivity can occur rapidly, even in one generation, and be accompanied by a decrease of fitness in the wild (Araki et al., 2007, 2009; Christie et al., 2012, 2016; Ford et al., 2016; Fraser et al., 2019; Thompson et al., 2018). Further, transmission of epigenetic parental effects, particularly DNA methylation, has also been suggested as a possible mechanism by which captive rearing could induce phenotypic changes that ultimately impact the fitness of individuals and (or) their offspring (Bonduriansky et al., 2012; see also Koch et al., 2023). Irrespective of the underlying cause, however, the ability of hatchery stocks to maintain variability in key ecologically‐relevant characteristics across generations may vary among species and populations, and be dependent on their maintenance practices (e.g. Araki & Schmid, 2010; Evans et al., 2014; Fraser et al., 2019; Hecht et al., 2015).

Different fish genotypes, often treated as sibling groups, strains or populations, may greatly vary in their average performance responses to environmental qualities (Hutchings, 2011; Sae‐Lim et al., 2016). Genotype‐by‐environment interactions (G × E) can manifest in two basic ways: either the amount of genetic variation differs (scaling effect) or the ranking of genotypes changes across different environments (Lynch & Walsh, 1998; Mulder & Bijma, 2005). In the latter case, the genotypes that perform well in one environment are not necessarily the best ones in other environments. The variation of reaction norms among genotypes provides information about the macro‐environmental sensitivity (or plasticity) and capacity of a population to adapt to environmental changes (Ghalambor et al., 2007; Hutchings, 2011; Sae‐Lim et al., 2016).

In nature, fish larvae and juveniles, including salmonids, typically experience very intense selection, and only a small fraction survive their first year of life (Cunjak & Therrien, 1998; Gibson, 1994). This differs fundamentally from the simple and benign conditions of a hatchery that allow a considerably higher subset of individuals to pass the most sensitive bottleneck phases. Due to the highly divergent selection regimes most of the genotypes produced and capable of living in captivity may perform poorly or even be lost in the wild (Glover et al., 2004; Weber & Fausch, 2003). Particularly in small populations, strong selection can deplete genetic variation before adaptation is possible (Fisher, 1930; Merilä & Sheldon, 1999). On the other hand, because sources of mortality typically vary in time and space, the genetic architecture of survival and its relationship with other traits will also vary significantly (Vehviläinen et al., 2008, 2012).

Captive breeding has been conducted for over 50 years to maintain the critically endangered landlocked Atlantic salmon (*Salmo salar* m. *sebago* Girard) population native to Lake Saimaa in Eastern Finland (Janhunen et al., 2022; Pursiainen et al., 1998). Due to the construction of hydroelectric dams in key rivers in the system, a complete natural life cycle of the Lake Saimaa salmon has not been possible since the late 1960s. Therefore, the population has been maintained in its original range by annual stockings of hatchery‐reared juveniles (mainly 2‐year‐old smolts), and by establishing new hatchery generations using wild‐caught spawners (Hutchings et al., 2019). Low post‐stocking survival of Saimaa salmon has further limited the numbers of adults available to support the captive breeding program, resulting in repeated genetic bottlenecks, and consequently this salmon strain is characterized by very low genetic diversity and a high degree of relatedness among individuals (Koljonen et al., 2002; Säisä et al., 2005; Tonteri et al., 2005). Low genetic diversity in Saimaa salmon is also linked to impaired foraging success and incidence of early developmental malformations (Primmer et al., 2003; Tiira et al., 2006). Moreover, a long hatchery history may have contributed to the apparent signs of domestication in behavioral traits related to exploration (boldness) and stress tolerance (Eronen et al., 2023). These factors may compromise the ability of the strain to tolerate and/or adapt to environmental changes and thus its return to the wild, despite recent habitat restoration efforts (Hatanpää et al., 2021; Leinonen et al., 2021). Thus far, there have been few experimental studies on possible early‐environment‐dependent selection patterns for salmonid genotypes (Crespel et al., 2013; Vasemägi et al., 2016).

In this study, we examined the parental factors influencing performance of Lake Saimaa salmon families during early life stages, first from fertilization to the late alevin (swim‐up) stage and then through the first summer in two environments. The experimental families produced with a factorial mating design were moved either to standard hatchery rearing or to a seminatural environment in artificial streams at the end of the alevin phase. At the age of 5 months, the sampled survivors from both environments were assigned to their parents by single nucleotide polymorphism (SNP) genotyping. Our aim was to assess how different environments select for salmon genotypes during the critical life stages, when most mortality takes place. Because selection is likely to favor different traits in the two environments studied, we predicted that the final representativeness of families would be inconsistent across the environments and might involve more heterogeneity under the seminatural conditions, where mortality is expectedly higher. Secondly, we also quantified the adaptive potential of progeny to divergent conditions by estimating genetic variation and the degree of G × E in growth traits of the fish across the environments. Further, we specifically examined whether G × E in juvenile growth resulted more from re‐ranking of genotypes or from heterogeneity of genetic variances between the environments.

## 2 MATERIALS AND METHODS

### 2.1 Gamete collection, fertilizations, and incubation

On 14 October 2021, the gametes of 12 females (year class 2015; total body length mean ± SD = 544 ± 32 mm) and 12 males (year class 2019; 223 ± 10 mm) were collected from captive Lake Saimaa salmon broodstocks maintained at Taivalkoski Aquaculture Station by the Natural Resources Institute Finland (Luke). Stripped eggs were stored in 1.5 L plastic airtight containers and stripped milt in 0.5 L plastic ziplock bags filled with pure oxygen. All the gametes were stored on ice and transferred to the Kainuu Fisheries Research Station in Paltamo (www.kfrs.fi) for fertilizations and sperm motility measurements, which took place the following day (15 October 2021).

The sperm activation trials (in ovarian fluids; see below) and fertilizations were conducted using a factorial 3 × 3 mating design, resulting in four half‐sib family blocks. Thus, the total number of matings was 36, and each pairwise fertilization (full‐sib family) was further replicated three times, forming 108 egg batches in total. The eggs from each female were split into nine portions using roughly similar numbers of eggs per batch (mean 159, range 146–172 eggs, *n* = 108 egg batches), which was determined based on female‐specific mean egg mass (weighted sample of eggs without ovarian fluid). The 108 individual fertilizations were performed in 0.2 L plastic cups by pipetting 200 μL of milt directly onto the eggs and then immediately activating the sperm with water.

**FIGURE 1:**
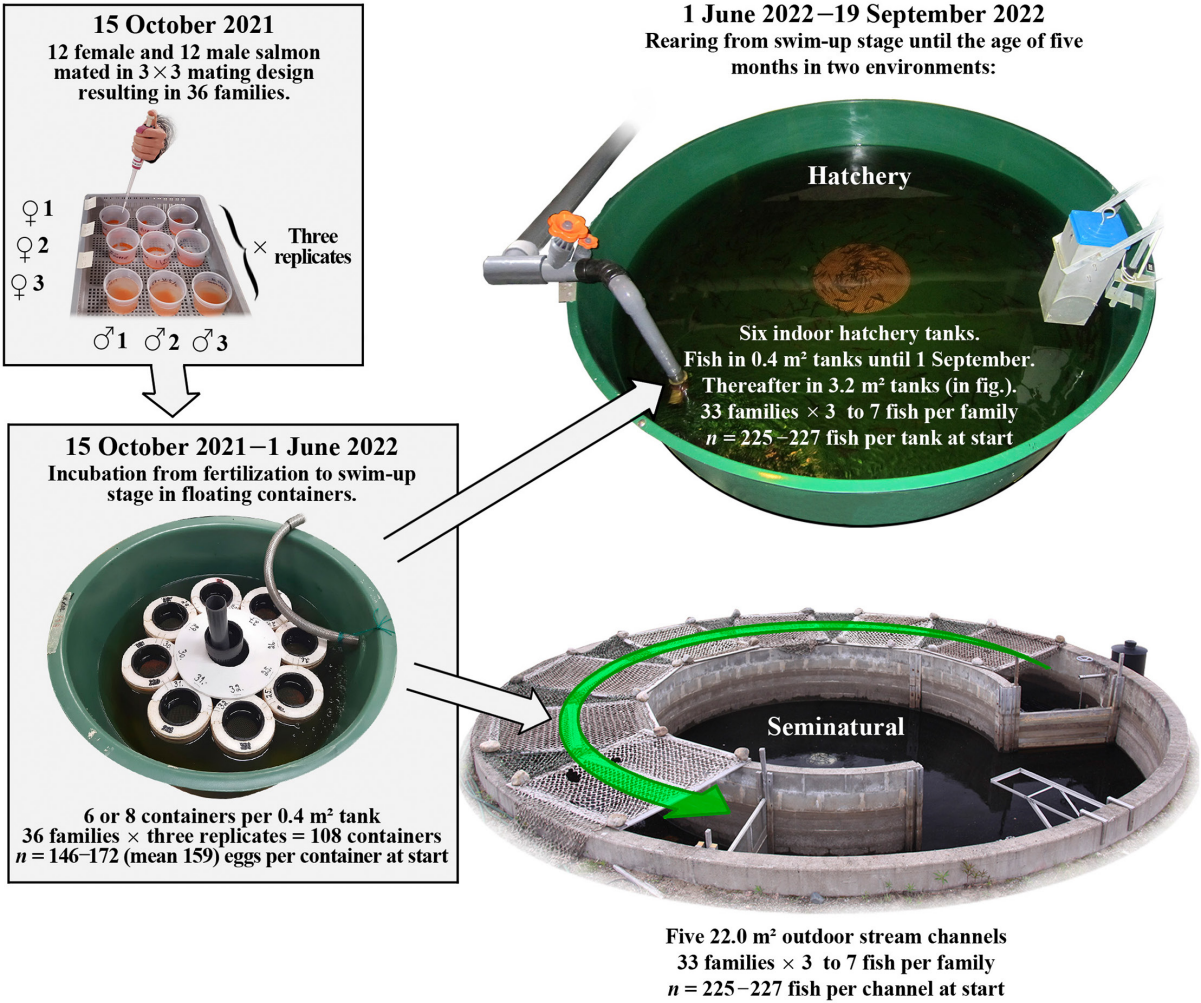
Overview of the experimental setup showing the experimental rearing units, and number of families and individuals in different phases of the study. The stream section of the seminatural channels (length 18 m), where the fish were kept (area and water flow direction indicated with a green arrow), was covered with camouflage netting and isolated from the central pool part (uncovered area) with fences.

The number of rearing units, families and fish individuals used in different phases of the study is summarized in Figure 1. Following fertilizations, the 108 egg batches were placed into separate plastic incubation cylinders (ø 10.2 cm) with a net bottom (mesh size 3 mm) (Eronen et al., 2021). The 108 floating cylinders circled slowly in groups of 8 or 6 units in 15 round 0.4 m^2^ tanks. The cylinders were randomized among the tanks by assigning full‐sib family replicates always to different tanks. Incubation temperature and oxygen content followed natural variation of the inflowing water from Lake Kivesjärvi (temperature range 0.3–9.6°C, oxygen range 6.3–12.3 mg L^−1^). Dead eggs and alevins were counted and removed from cylinders weekly in order to prevent the occurrence and spread of oomycete (*Saprolegnia* sp.) infections. At the eyed‐embryo egg stage, embryos were mechanically disturbed by pouring each egg batch into a plastic cup (22–24 February 2022). Thereafter the eggs that were unfertilized or contained an aborted embryo turned white and were removed (mean ± SD survival = 53.4 ± 24.3%, range 0.6–83.4, *n* = 36 full‐sib families). Before hatching, all egg batches were moved into cylinders with a smaller mesh net (1 mm) to prevent alevins from escaping (29–30 March 2022). Then, 81 cylinders with more than 50 eggs were further divided to two or three replicates to avoid overcrowding and the consequent blockage of water. The hatching period lasted until 2 May 2022. Pre‐ and post‐hatching survivals were determined for each incubation unit based on the proportions of live eyed embryos (25 February 2022) and swim‐up alevins (31 May 2022), respectively. Because these two survival measures were highly positively correlated (Pearson's *r* = 0.992, *n* = 108 incubators), they can be interpreted as equivalent traits involving variation due to fertilization success and later developmental failures. For this reason, only the swim‐up stage was taken into account in further analyses.

### 2.2 Sperm activation trials

For each male, the motility recordings of milt samples were separately performed in hatchery water (control activation) and in each of the three ovarian fluid samples used in fertilizations. In externally fertilizing fish, females are known to alter sperm behavior by their ovarian fluid, which is released along with the eggs (Alonzo et al., 2016; Zadmajid et al., 2019). Generally, ovarian fluid improves sperm swimming performance compared to a water‐only environment but may also act as a selective medium for fertilizing males (cryptic female choice) (Zadmajid et al., 2019).

The sperm activations were conducted using a B/W CCD camera (capture rate of 60 frames s^−1^) mounted to a negative phase contrast microscope (100 × magnification) (total *n* = 48 recordings). After vortexing the sperm samples for 5 s, 0.1 μL of milt from each of the 10 males was pipetted to 20 μm Leja 2‐chamber microscope slides (Leja, Nieuw‐Vennep, the Netherlands), and then sperm motility was activated by adding 3 μL of either water or ovarian fluid‐water (1:1) mixture. Thereafter, percentage of static sperm cells and sperm curvilinear swimming velocity were determined 10 s post‐activation using computer‐assisted sperm analysis (Integrated Semen Analysis System, ISAS version 1.2 Proiser, Valencia, Spain).

### 2.3 On‐growing experiment

On 1–2 June 2022, shortly before the alevins had completely absorbed their yolk reserves (464 degree‐days from fertilization), random samples of individuals from 33 families were moved to six round indoor tanks made of green plastic and to five circular seminatural outdoor concrete channels containing a gravel bed bottom (*ø* ~30–80 mm, depth ~50 cm) interspersed with ~50 bigger boulders (~10 × 15 cm). At this stage, all half‐sib families of one female had to be excluded from the experiment due to high mortality at the pre‐hatching stage. Out of 33 full‐sib families, 31 families contained a full set of 84 alevins divided equally into all hatchery tanks and seminatural channels (7 alevins per family per tank/channel). In two families the number of alevins varied between 3–4 and 5–6 per tank. The total number of alevins among the 33 full‐sib families was 2710 at the beginning of the experiment (*n* = 225–227 fish per tank).

The six indoor hatchery tanks had a bottom area of 0.4 m^2^ and a water depth of 32 cm (~130 L). Water flow rate in the tanks was 0.3–0.4 L s^−1^ and temperature was 9.5–10.3°C at the beginning, increasing later to a maximum of 17.7°C in the summer. In the hatchery treatment the fish were provided with conventional husbandry, which involved regular cleaning of tanks with a brush, daily feeding with commercial dry feed (Raisioagro Ltd, Raisio, Finland) by automatic feeders, removal of dead fish and temporary supply of additional oxygen to the incoming water during the warmest period (starting from 18th July). The amount of feed provided was adjusted based on water temperature and estimated tank biomass. After start‐feeding (with 0.2/0.5 mm Veronesi VITA), pellet size was also changed gradually according to the growth of fish following the manufacturer's recommendations. A natural photoperiod corresponding to local conditions was maintained throughout the on‐growing period. On 1st September, the indoor hatchery environment fish were moved to six larger round indoor fiberglass tanks for the rest of the experiment (bottom area 3.2 m^2^, water depth 20 cm, water flow 0.6 L s^−1^). The rearing lots were unchanged (*n* = 199–220 fish per tank).

The five replicate seminatural stream channels covered three quarters of an entire circle (outer circumference of the experimental sections 18.0 m, width 1.5 m, mean water depth ~20 cm). Mean water surface flow in the stream channels was 9 and 4 cm s^−1^ measured at 4 and 19 m from the inlet pipe, respectively, and water temperature was 8.7°C at the time of fish transfer. As in the study of Rodewald et al. (2011), the fish were not provided any commercial feed within the channels but were completely dependent on the ability to forage on natural drift or benthic prey. The channels were covered for the most parts with camouflage netting, which provided the fish with shading and eliminated the mortality caused by avian predators (gulls).

The experiment was terminated on 19–21 September, when samples of live one‐summer‐old salmon were randomly collected from each hatchery tank and seminatural channel (total *n* = 835 fish). The sample size was 80 fish for each hatchery tank and three outdoor channels, whereas for two outdoor channels the number of sampled fish was 55 and 60. Variation in the sample sizes within two channels was due to accidental escape of some fish to the central pool part of the rearing units. The escapes had occurred in the very early phase of the experiment, because the mesh size of the sieve net installed between the stream and pool parts of each tank was only 2 mm. All live fish found in the pool side of the tanks were omitted from sampling. As the sieve nets in all tanks were identical, the remaining fish are considered to uniformly represent habitat selection that had occurred after the first week of the experiment. The actual mortalities, however, cannot be reliably reported for the on‐growing period in the seminatural environment. All sampled fish were sedated using benzocaine (40 mg L^−1^) and their total body length (BL, in mm) and mass (BM, with a resolution of 0.1 g) were measured. In addition, a small sample clipped from the lower tip of each fish's caudal fin was preserved in 99.8% ethanol for later parentage analyses. Because the sampled fish were kept for a hatchery broodstock, the parentage analyses also serve their later kinship management.

The adjusted condition factor (CF) was calculated for each sampled fish using the equation: CF = 100 × (BM (g) × BL (cm))^−*b*^, where *b* is the slope of a nonlinear regression of BM on BL from the environment‐specific pooled data of individual measurements; 3.06 for the hatchery and 3.10 for the seminatural environment.

The study compiled with the animal care legislation of Finland and did not involve procedures that would have required a permit issued by the national Animal Experiment Board (ELLA).

### 2.4 Parentage assignment

Parentage analysis was performed using caudal fin tissue samples to assign the sampled individuals to their respective full‐sib families. DNA was extracted using Lucigen QuickExtract™ solution (Lucigen, Co.—LGC, Biosearch Technologies, Ltd., Middletone, WI, USA). The multiplex PCR panel included 152 SNPs loci originally identified to be polymorphic in another Finnish population of Atlantic salmon (the Teno River; for more details, see Aykanat et al., 2016), 49 of which were polymorphic in the studied population. Sequencing was performed at the DNA Sequencing and Genomics Laboratory (BIDGEN) laboratory at the Institute of Biotechnology, University of Helsinki, using the Illumina NextSeq500 platform and 1 × 150 bp sequencing (Illumina, Inc., San Diego, CA, USA).

Parental assignment was performed using the software snppit (https://github.com/eriqande/snppit). The assignment accuracy was assessed by simulation of offspring genotypes. The actual parents (12 sires and 12 dams) were genotyped prior to offspring genotyping, and for every possible sire‐dam combination, the genotype of one offspring was simulated. Identical‐by‐descent (IBD) values for all individual pairs and Mendelian errors for all individual trios were calculated. Based on the highest IBD value, 90% of simulated offspring were assigned to their correct parents. When considering Mendelian errors, all simulated offspring were assigned correctly. However, Mendelian errors were not simulated.

### 2.5 Statistical analyses

All data analyses were performed in SAS 9.4. (SAS Institute, Cary, NC, USA). Alternative statistical models with different composition of covariates and random variance parameters were compared, and the final model was selected based on the estimatability or significance of variances (likelihood ratio test), and on Akaike's information criterion. First, parental influence on offspring survival from fertilization to the late alevin (swim‐up) stage (15 October 2021–31 May 2022) was evaluated separately using generalized linear mixed models (GLMM, Proc GLIMMIX in SAS), with logit link function and Laplace approximation method. Here, individual incubation cylinders (*n* = 108) were statistical units, and the response followed a binomial *y*/*n* form, where *y* = number of live swim‐up alevins and *n* = total number of eggs at the beginning of the experiment. The final models included random intercepts for females (covariation among maternal half sibs), males (covariation among paternal half sibs) full‐sib families (parental interaction) and incubation tank (*n* = 15). We also tested whether the sperm motility variables determined for each male predicted early survival as fixed covariates. The sperm traits were included in the models as variable pairs, depending on whether they were measured in water (one value per male per trait) or for each ovarian fluid—milt combination (three different values per male per trait).

For the on‐growing period, the sample frequencies of one‐summer‐old fish per family (*y*) in relation to initial number of alevins (*n*) per family was used as a binomial response for each experimental tank and channel. Consequently, a GLMM was first run for both environments (hatchery or seminatural) together using full‐sib family and environment × full‐sib family as random effects. Thereafter, to estimate among‐family variation independently for each environment, separate GLMMs were run by defining full‐sib family as a random effect. For the seminatural environment, the model also contained a significant full‐sib family × tank interaction effect which corresponded to a residual term. Inclusion of sire variance led to estimation problems in all models, due to observed zero variance. Dam identity was also excluded from each model, because its variance was confounded with that of the full‐sib family, and both terms could not be incorporated in the same models due to estimation problems of the other (full‐sib) variance. Relationships of random solutions (best linear unbiased predictions, BLUPs) obtained from environments‐specific models (on‐growing) were estimated for full‐sib families using Spearman's rank correlation analysis. Similarly, the family survival estimates at the swim‐up stage were correlated with those obtained at the end of the experiment in both on‐growing environments. To further visualize the degree of sire‐ and dam‐family variation and re‐ranking of sires and dams across environments in offspring (re)sampling probability, the estimates were calculated for different environments using the model which included interaction terms between environment and sire, and between environment and dam.

For fish growth traits (BL, BM, and CF), the differences of means between the environments were first compared using a linear mixed‐effects model (Proc MIXED) with restricted maximum likelihood estimation method (REML). Here, environment was used as a categorical fixed effect, and tank (or channel), nested within environment, was a random effect. Separate residual variances were estimated for each environment group, as they improved the model fit for each trait. In addition, standard error and degrees of freedom for the tests of the fixed effect were calculated using the method by Kenward and Roger (1997). Genetic and phenotypic (co)variation in the two environments was estimated for all growth traits using three separate 4‐trait animal models (two trait pairs together per model) and applying REML and average information methods (DMU 6.0 software; www.dmu.agrsci.dk). Each model included experimental tank or stream channel as a fixed effect and the random genetic animal effect linked to the pedigree (parents). In the genetic model, the fixed effect corrects for any environmental variation caused by the tanks or channels when all families are present in each of them. Residual covariance was set to zero for each trait pair recorded in different environments. Heritability of each growth trait was quantified as $h^{2}=\sigma_{g}^{2}{\left(\sigma_{g}^{2}+\sigma_{e}^{2}\right)}^{-1}$, where $\sigma_{g}^{2}$ is genetic variance and $\sigma_{e}^{2}$ is residual variance. Although $\sigma_{g}^{2}$ is assumed to be mainly due to additive genetic effects, the potential influence of dominance and epistasis cannot be excluded. Asymptotic standard errors for the genetic parameters were computed based on a Taylor series approximation. The genetic correlations were calculated for each growth trait pair (Falconer, 1952): $r_{g}={\mathrm{COV}}_{g1,g2}/\sqrt{\sigma_{g1}^{2}\sigma_{g2}^{2}}$, where ${\mathrm{COV}}_{g1,g2}$ is the genetic covariance estimate of two traits. Lastly, heterogeneity of genetic, residual and phenotypic variation in all growth traits was compared between the environments by calculating genetic $\left({\mathrm{CV}}_{g}=100\times \sqrt{\sigma_{g}^{2}}÷\bar{X}\right)$, residual $\left({\mathrm{CV}}_{e}=100\times \sqrt{\sigma_{e}^{2}}÷\bar{X}\right)$ and phenotypic $\left({\mathrm{CV}}_{p}=100\times \sqrt{\sigma_{p}^{2}}÷\bar{X}\right)$ coefficients of variation (%), where $\bar{X}$ is the phenotypic mean of each trait in the population. The ${\mathrm{CV}}_{g}$ was used to estimate the degree of genetic variation across environments because it is standardized by the mean differences of traits across environments (Houle, 1992). CVs are also useful for assessing whether a high heritability of a trait results from high genetic variation or from low residual variation.

## 3 RESULTS

### 3.1 Survival from fertilization to the swim‐up stage (hatchery environment)

The mean survival among families from fertilization to late alevin (swim‐up) stage in the hatchery environment (15 October 2021–31 May 2022) was 48.7% ± 22.6 SD (range of 0.4%–77.8%, *n* = 36 full‐sib groups). According to the GLMM, both parental identities, their interaction (i.e. full‐sib family) and incubation tank had statistically highly significant effects on early offspring survival, the most variation being due to dams (Table 1). Irrespective of the activation medium (water or ovarian fluid), no sperm motility trait (percentage of static sperm cells and curvilinear swimming velocity 10 s post‐activation) predicted offspring viability at the swim‐up stage and were thus excluded from the final model (Table 1).

**TABLE 1:** Estimates of parental effects and sperm motility traits (covariates, measured per male either in water or ovarian fluid activations) on landlocked salmon early survival, determined from fertilization to the swim‐up alevin stage in hatchery incubation.

Parameter	*n*	Estimate ± SE	*p*‐Value	% of total variance
Dam	12	1.546 ± 0.686	<0.001	27.2
Sire	12	0.721 ± 0.345	<0.001	12.7
Dam × sire	36	0.090 ± 0.042	<0.001	1.6
Tank	15	0.034 ± 0.016	<0.001	0.6
Residual		*π* ^2^/3		

	df	Estimate ± SE	*F*‐value	*p*‐Value
Static sperm (%)				
In water	1, 58	0.062 ± 0.042	2.18	0.145
In ovarian fluids	1, 57	0.012 ± 0.038	0.09	0.764
Sperm velocity				
In water	1, 58	−0.040 ± 0.024	2.82	0.098
In ovarian fluids	1, 57	0.016 ± 0.015	1.16	0.285

### 3.2 Representativeness of families in two rearing environments

**FIGURE 2:**
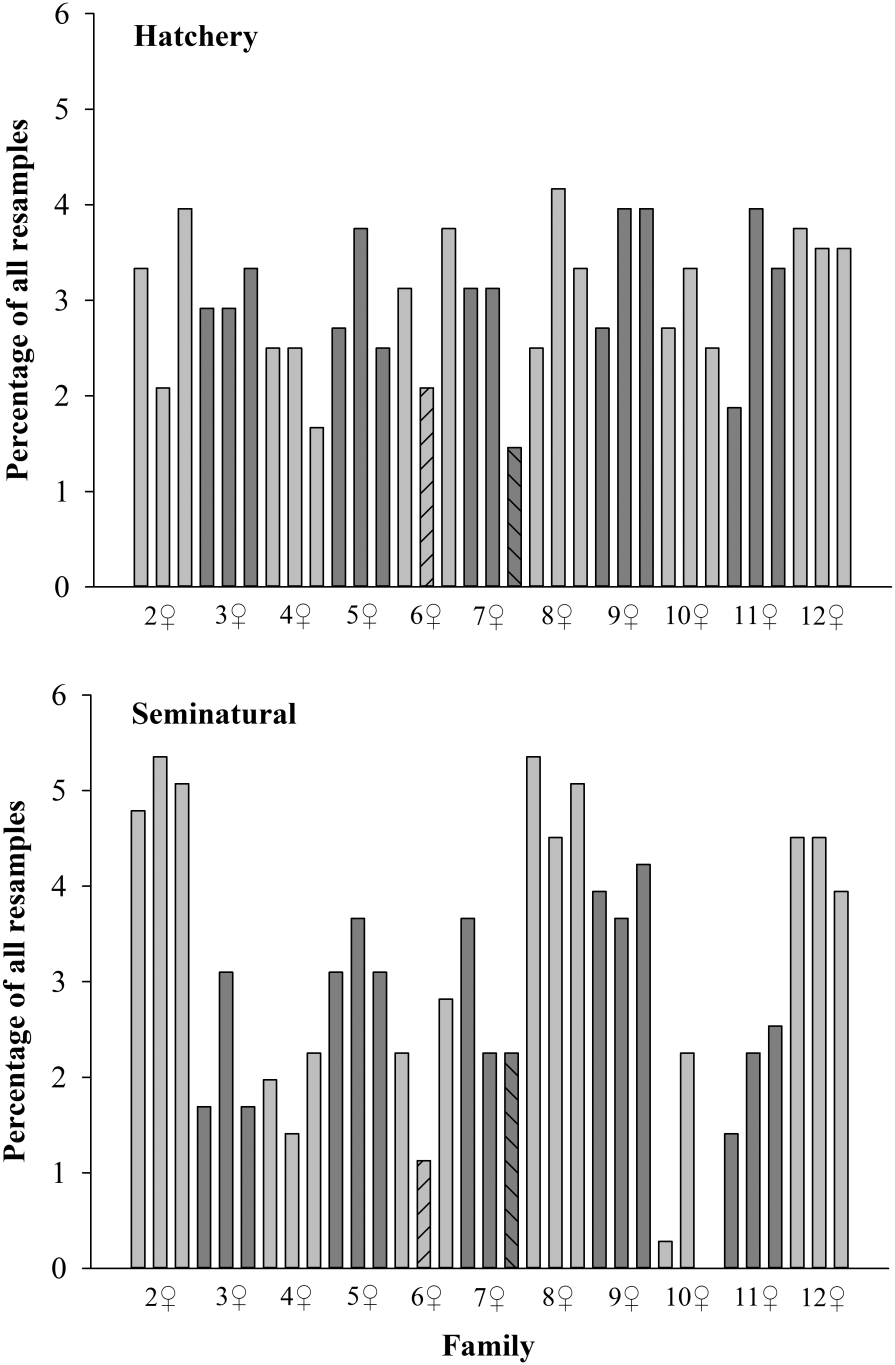
Percentual distribution of one‐summer‐old landlocked salmon families sampled from two rearing environments (total *n* = 480 fish in hatchery and 355 fish in seminatural environment). Each female was mated with three males. Two full‐sib groups having a smaller number of fish at the beginning of the experiment (19 + 19 alevins in family 6♀ × 5♂ and 30 + 29 alevins in family 7♀ × 9♂) are shown in the graph with dashed bars. For all other families, the initial number of fish was 42 + 35 in the hatchery + seminatural environment, respectively.

In indoor hatchery tanks, the mean survival from swim up to the age of 5 months was 88.7% (range 81.8%–95.6%, *n* = 6 tanks), and all families were present at the end of the on‐growing period (1 June–21 September 2022). The mean sample proportion per hatchery‐reared family, relative to their initial sample sizes, was 35.7% (range 19.0%–52.6%, *n* = 33 families) (Figure S1). In the seminatural environment, the corresponding proportion per family was on average 31.2% (range 0.0%–54.3%). One of the families (♀10 × ♂12) was missing and only one offspring was found for another half‐sib group of the same dam (♀10 × ♂10) (Figure 2; Figure S1). The number of families found among the end samples varied between 29–32 in the hatchery tanks (average 30.5 families, *n* = 6 tanks) and between 25 and 31 in the seminatural channels (average 27.8 families, *n* = 5 channels). Overall, the occurrence of different families clearly showed higher variation in the samples collected from the seminatural environment than from the hatchery environment (Figure 2).

**FIGURE 3:**
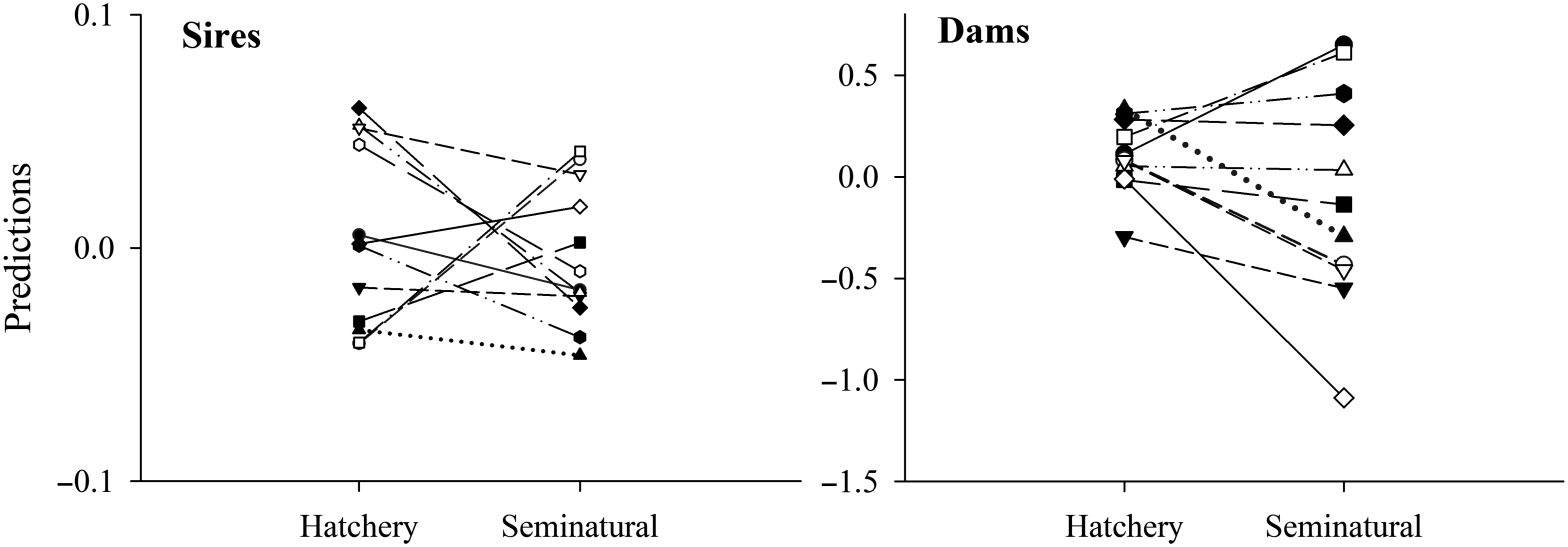
Sire and dam estimates (on logit scale) for the likelihood of one‐summer‐old salmon to become sampled after the first summer in hatchery and seminatural rearing environments. Separate lines connect the estimates for each parental individual between the two environments.

When the GLMM was run for combined data, close‐to‐significant variation was found among the full‐sib families (estimate ± SE = 0.066 ± 0.051; likelihood ratio test: *χ* ^2^ = 1.81, df = 1, *p* = 0.090), whereas the family × environment interaction was statistically significant (0.098 ± 0.059; *χ* ^2^ = 5.87, df = 1, *p* = 0.008). Thus, the likelihood of different full‐sib families to be sampled differed across the environments. When the GLMMs were run separately for each environment, the variance among full‐sib families was not significant in the hatchery environment (estimate ± SE = 0.004 ± 0.028; *χ* ^2^ = 0.03, df = 1, *p* = 0.436). Instead, full‐sib families differed significantly in their sampling probabilities in the seminatural environment (estimate ± SE = 0.389 ± 0.158; *χ* ^2^ = 19.28, df = 1, *p* < 0.001). The cross‐environment correlation of family predictions obtained from separate models was positive and close to statistical significance (*r*_s_ = 0.341, *p* = 0.052, *n* = 33 full‐sib families). Increased heterogeneity of parental estimates in the seminatural environment was considerably more apparent for dams than for sires (Figure 3). Family survival prediction until the swim‐up stage was not significantly correlated with the family probability predictions obtained from the on‐growing in standard (*r*_s_ = 0.188, *p* = 0.295, *n* = 33) or seminatural environment (*r*_s_ = 0.092, *p* = 0.612).

### 3.3 Growth variation in two environments

Body length and mass were, on average, significantly higher (BL: *F*_1,8.54_ = 24.18, *p* = 0.001, BM: *F*_1,8.23_ = 23.76, *p* = 0.001) in salmon juveniles reared in the seminatural environment (BL: 80.5 ± 1.1 mm, BM: 4.7 ± 0.2 g; least square mean ± SE) compared to the hatchery environment reared individuals (BL: 73.3 ± 1.0 mm, BM: 3.6 ± 0.1 g). However, hatchery tank‐reared individuals had higher mean CF (0.790 ± 0.004), compared to the fish reared in the seminatural environment (0.703 ± 0.004; *F*_1,9.06_ = 227.40, *p* < 0.001).

**FIGURE 4:**
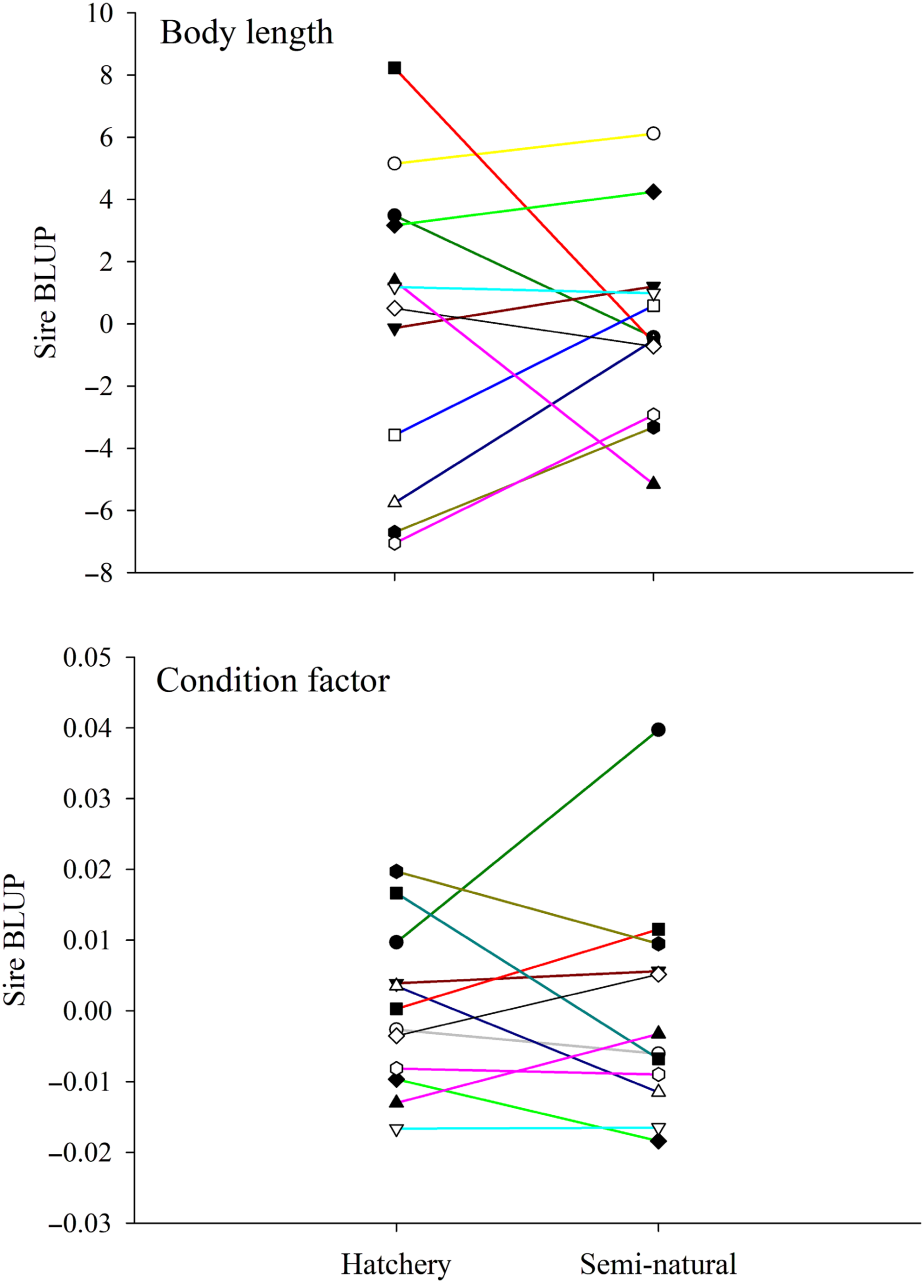
Genetic values (best linear unbiased predictions [BLUP]) of sires for body length and condition factor across rearing environments. In each graph, separate lines connect the estimates for each parent between the two environments.

The *h* ^2^ estimates for body size traits (BL, BM) were moderately high and of the same magnitude in the hatchery (0.62, 0.68) and seminatural environments (0.69, 0.62) (Table 2). In contrast, the *h* ^2^ of CF was relatively low in both environments (0.16–0.20). Both body size traits showed positive genetic correlation (*r*_g_ = 0.67–0.69) across environments (Table 2). The *r*_g_ estimate of CF was also positive, but being associated with large standard error, was potentially non‐significant. Interestingly, CF in hatchery showed a moderate positive genetic correlation of 0.54 with body mass in the seminatural environment (Table 2). Figure 4 shows reaction norms for body length and CF at the sire level across the environments. For all growth traits, the variation among hatchery tanks or stream channels was of minor importance.

**TABLE 2:** Estimates of heritability (*h* ^2^, diagonal), and genetic (*r*_g_, below diagonal) and phenotypic (*r*_p_, above diagonal) correlations for body length (mm), mass (g) and condition factor of one‐summer‐old landlocked salmon grown in hatchery and seminatural environments. Standard errors of the estimates are given in parentheses. All *h* ^2^ and *r*_g_ estimates significantly different from zero and from unity are highlighted in bold font (0 and 1 not within estimate ± 1.96 SE).

Trait	Length_hatchery_	Length_seminatural_	Mass_hatchery_	Mass_seminatural_	Condition_hatchery_	Condition_seminatural_
Length_hatchery_	*h* ^2^ = **0.618** (0.114)	ne	0.968	ne	−0.188	ne
Length_seminatural_	**0.665** (0.114)	*h* ^2^ = **0.694** (0.127)	ne	0.965	ne	−0.189
Mass_hatchery_	**0.994** (0.003)	**0.675** (0.111)	*h* ^2^ = **0.678** (0.118)	ne	0.010	ne
Mass_seminatural_	**0.679** (0.113)	**0.992** (0.004)	**0.694** (0.108)	*h* ^2^ = **0.620** (0.122)	ne	0.017
Condition_hatchery_	0.101 (0.209)	**0.536** (0.163)	0.193 (0.202)	**0.542** (0.165)	*h* ^2^ = **0.198** (0.060)	ne
Condition_seminatural_	0.096 (0.232)	−0.139 (0.236)	0.180 (0.227)	−0.014 (0.242)	0.363 (0.241)	*h* ^2^ = **0.162** (0.062)

Abbreviation: ne, non‐estimable—not recorded from same individuals.

The growth traits differed in terms of both CV_g_, CV_e_ and CV_p_, indicating the presence of some degree of heterogeneity in genetic, residual and phenotypic variation across the environments (Table 3). Especially the phenotypic variation (CV_p_) of BM increased in the seminatural environment compared to hatchery rearing. This was more a result of increased residual (CV_e_) than genetic variation.

**TABLE 3:** Phenotypic variance ($\sigma_{p}^{2}$), and genetic (CV_g_), residual (CV_e_) and phenotypic (CV_p_) coefficients of variation (%) in body length, mass and condition factor of one‐summer‐old landlocked salmon reared in two different environments.

Trait	Environment	$\sigma_{p}^{2}$	CV_g_	CV_e_	CV_p_
Length (mm)	Hatchery	70.730	9.1	7.1	11.5
	Seminatural	107.894	10.8	7.2	13.0
Mass (g)	Hatchery	1.446	27.6	19.0	33.5
	Seminatural	3.549	31.8	24.9	40.4
Condition	Hatchery	2.291^−3^	2.7	5.4	6.1
	Seminatural	2.888^−3^	3.1	7.0	7.6

## 4 DISCUSSION

Early rearing conditions can strongly modify the performance of fish juveniles, potentially inducing fitness‐related variability among genotypes (e.g., Hatanpää et al., 2020; Janhunen et al., 2010, 2016; Kortet et al., 2019). Genotype‐by‐environment interaction thereby plays an important role in preserving genetic variation of quantitative traits in natural and captive populations (Gillespie & Turelli, 1989; Mackay et al., 2009). Very few earlier studies have experimentally tackled this topic in salmonids, however (Crespel et al., 2013; Vasemägi et al., 2016). Here, we investigated environment‐dependent selection among Lake Saimaa landlocked salmon juveniles until the age of 5 months. We found higher survival (88.7%) and clearly more even distribution (representation) among individuals reared in standard hatchery conditions after the first summer compared to individuals from the same families reared in seminatural conditions. A major part of the increased survival differences among sibling groups in the seminatural environment appeared to be due to dams, which emphasizes maternal contribution to offspring early success under temporally or spatially more food‐limited conditions (Einum, 2003; Einum & Fleming, 1999). Environmental differences also influenced growth traits (body size and condition) of the study individuals, but our findings interestingly indicate that there was substantial additive genetic variation in these phenotypic responses, too. The unexpectedly high heritability levels in growth suggest that the hatchery strain is still capable of responding to selection in both natural and artificial environments. Furthermore, the salmon genotypes that grew well in hatchery rearing appeared to be mainly those that also grew well with natural food.

### 4.1 Survival selection

Variation in survival from fertilization to the swim‐up stage involved mainly maternal variation, but we also detected a significant sire variation as an indication of possible additive genetic effects. The role of epigenetic inheritance (such as DNA methylation in eggs and sperm) cannot be completely ruled out as a potential mechanism explaining part of the observed maternal or paternal influence on early survival (reviewed by Koch et al., 2023). Yet, as offspring performance was not compared between differently treated parents (see e.g. Evans et al., 2014), disentangling genetic and non‐genetic transgenerational variations requires alternative experimental designs. Regardless, it seems apparent that the among‐sire variation was not linked to differences in sperm motility (measured either in water or as pairwise ovarian fluid activations) and thus not directly linked to differences in male fertilization capacity. The ontogenetic decrease in predominant maternal effects coupled with concurrent increases in additive and non‐additive genetic effects has been well documented in salmonids, including Atlantic salmon (Heath et al., 1999; Houde et al., 2013, 2015; Perry et al., 2004). Further, maternal effects have been specifically linked to varying metabolic rates and energetic needs of salmonid offspring at emergence (Régnier et al., 2010), as well as to variation in later behavior, when the social dominance of offspring is affected by the hormonal state of the female (Eriksen et al., 2011).

Maximizing genetic diversity originally preserved in hatchery strains should be prioritized in supportive breeding programs aiming to promote the conservation and productivity of wild salmonid stocks (Withler et al., 2014). Nevertheless, the avoidance of artificial selection and consequent genetic changes remain an obvious challenge (Gamble & Calsbeek, 2023; Neff et al., 2011; Pasquet, 2019; Ryman & Laikre, 1991). In practical broodstock management, families of salmon are often combined already before hatching (at the eyed‐egg stage), and thus their survival differences can no longer be assessed over the course of on‐growing. When we took the same number of alevins from the experimental families to standard hatchery rearing, members of all families were still present after the first summer of growth. Compared to seminatural rearing, the frequency distribution of hatchery‐reared families also remained less variable; excluding one full‐sib group with fewer offspring at the beginning of the on‐growing phase, the family shares varied between 1.7% and 4.2% (Figure 2). However, only one full‐sib family out of 33 was completely missing from the final samples of the seminatural environment, and only one fish was obtained from another half‐sib group of the same dam; range of family shares 0.0%–5.4% of all sampled fish (Figure 2). Thus, at least some individuals of most families were able to find and utilize variable natural food and survive the first summer.

The absence of sire variation in offspring sampling probabilities suggests that survival during the on‐growing period presumably did not involve significant additive genetic variation in either of the environments tested. We instead found that the increased heterogeneity in the presence of salmon sib groups under seminatural conditions was also largely associated with dam (and non‐additive genetic) variation, indicating the environment‐dependent influence of mothers (and parental combinations) on offspring survival during fry‐fingerling phases. At the full‐sib family level, however, survival until the swim‐up stage was not correlated with later performance under hatchery rearing nor in seminatural conditions. Although variation in environmental conditions may often affect the expression of female‐mediated effects (Mousseau & Fox, 1998; Räsänen & Kruuk, 2007), dam‐by‐environment interactions have gained much less attention than G × E in evolutionary research (e.g. Vega‐Trejo et al., 2018). In salmonids, fitness variation caused by maternal effects is known to play a crucial role under natural and particularly under poor growth conditions, where large egg size provides a selective advantage for juveniles (Einum, 2003; Einum & Fleming, 1999). In the seminatural channels, where food is scarce and spatially distributed, a good buffering capacity against starvation is a particularly important trait. Most mortality occurred presumably soon after the alevins had been released to the stream channels and switched to external feeding. Overall mortality over the course of the experiment can, however, involve many factors that do not share a common genetic determination. Although our results conform to the general perception that survival as a fundamental component of fitness exhibits low or close to zero heritability (Merilä & Sheldon, 1999; Mousseau & Roff, 1987; Price & Schluter, 1991), consistent differences among genotypes may nevertheless occur across environments and cohorts (Vehviläinen et al., 2008). We can also expect that the stronger selection observed within our seminatural environment improved, on average, the adaptation of the surviving progeny to natural conditions, in relation to hatchery rearing.

### 4.2 Genetic (co)variation in growth

Surprisingly, the salmon juveniles reared in seminatural channels attained, on average, larger body size, whereas phenotypic size variation (both absolute and relative) became lower and CF higher in hatchery rearing. The larger mean size of the fish in the seminatural environment indicates good food supply, which was probably influenced by the lower density of the fish, compared to hatchery rearing. Environmental differences in body condition (length‐mass relationship) indicate that hatchery‐reared fish developed more robust body shape than those grown in the seminatural streams. This reasoning conforms to the previous finding on the same strain, where semiwild fish (stocked as alevins to a natural stream) developed a more slender body form compared to hatchery‐reared fish (Hatanpää et al., 2020).

Despite small effective population size and low overall genetic diversity, our heritability estimates for body size traits (*h* ^2^ = 0.62–0.69) were significant and clearly higher than the median values of 0.29–0.32 reported from a meta‐analysis of heritability for size‐at‐age across salmonid species (Carlson & Seamons, 2008). Further, especially the marked genetic coefficient of variation for body mass (~30%) supports the notion of the presence of substantial additive genetic variation in growth responses across the environments. The high levels of additive genetic variation suggest that both research environments maintained genotypes of variable growth, and, thus, artificial selection for juvenile growth has not been strong within the studied strain. Supporting this reasoning, an earlier study by Saikkonen et al. (2011) found no evidence that a standard hatchery environment would have induced directional selection in the growth of salmon juveniles from the same strain. In contrast, the juveniles grown under seminatural conditions were under weak directional selection against large body size (Saikkonen et al., 2011). Population bottlenecks have been found to induce an immediate increase in additive genetic variation and heritability of morphological and life history traits, presumably as a result of “converted” epistatic and dominance effects (van Buskirk & Willi, 2006; Willis & Orr, 1993). In brook charr (*Salvelinus fontinalis*) domestic and inbred hatchery strains have been found to exhibit considerably higher heritability in body length and mass than genetically more diverse and outbred strains (Crespel et al., 2013; Varian & Nichols, 2010). If hatchery selection for growth traits has been limited, additive variation could be expected to remain high after the bottleneck and may thereby explain the prominent *h* ^2^ estimates observed also in the Saimaa salmon strain.

Earlier evidence collected from various animal taxa suggested that environmentally induced variation tends to increase under unfavorable conditions, while the proportion of heritable variation decreases (Charmantier & Garant, 2005; Hoffmann & Merilä, 1999). However, in a more recent meta‐analysis, where the changes in mean‐standardized variances (CVs) were compared between benign and stressful environments, a significant positive effect of highly stressful conditions on additive and residual variation was found in life‐history traits, but not in morphological traits (Rowiński & Rogell, 2017). Supporting the latter view, we did not find that the amount of additive genetic variation in growth traits would have markedly changed across the environments (changes in CV_g_ 0.2%–4.2%‐units). For each growth trait, both genetic and residual coefficients of variation became even larger under seminatural conditions, though neither of the environments studied can perhaps be considered particularly unfavorable or stressful for salmon juveniles. Because CV_g_ describes the propensity of the trait to respond to selection, that is, its evolvability (Hansen et al., 2011; Houle, 1992), we can assume that the adaptive potential of growth remained high within the strain irrespective of the environment tested.

Although increased phenotypic growth variation in the seminatural environment was partly due to increased additive genetic variation, the scaling effect was probably a less significant source of G × E interaction than genotype re‐ranking. In this study, the influence of rearing environment on genotype re‐ranking in body size (*r*_g_ = 0.67–0.69) was of similar magnitude as reported for growth traits of fishes in conventional aquaculture settings (mean *r*_g_ = 0.70, 11 fish species; Sae‐Lim et al., 2016). The G × E interaction is generally considered biologically significant when genetic correlation is lower than 0.8 (Robertson, 1959). Hence, although body size had a clear positive genetic association between the environments, growth performance in the hatchery did not consistently predict genetic growth potential under more natural conditions. In other words, some genotypes with a slow growth in hatchery tanks seemed to react positively to the environmental change, i.e., growing faster under seminatural conditions, and vice versa. Vasemägi et al. (2016) recognized divergent quantitative trait loci (QTL) that control the growth of juvenile salmon in hatchery versus wild environments. Our multivariate analyses interestingly showed that not only size traits were positively genetically correlated with each other between the environments tested, but also high body condition in the hatchery was positively linked to rapid growth in the seminatural environment (*r*_g_ = 0.54).

Although the seminatural environment in our study challenged the viability of fish by forcing them to acquire natural food, an essential mortality factor, predation, was missing. In general, maladaptive behavioral changes due to artificial rearing environments, such as decreased predator avoidance are believed to be a major factor explaining the low post‐stocking survival rates of hatchery fish (Alioravainen et al., 2018; Brignon et al., 2017; Jackson & Brown, 2011; Näslund, 2017; Nickelson, 2003; Zhang et al., 2023). A recent study by Eronen et al. (2023) showed that Saimaa salmon juveniles exhibited, on average, more explorative behavior in a novel environment and were more stress‐tolerant than conspecifics from a genetically more diverse anadromous strain. The authors concluded that the observed behavioral differences between the strains may be related, at least in part, to higher level of domestication of the Saimaa salmon. The relatively high genetic association for growth between our study environments could be partly due to predator‐free conditions that consistently favored the most active and bold genotypes. However, the possible fixation of behavioral features reported by Eronen et al. (2023) might impose considerably stronger selection and G × E under natural conditions where the fish are susceptible to predation.

Finally, we note that unequal (smaller) sample sizes in some families combined with relatively low family number might have impacted the accuracy of genetic parameter estimation in growth traits. The use of a factorial mating design, on the other hand, improved our ability to evaluate genetic (co)variances, compared to a situation where a full‐sib (single‐pair) or nested design would have been used (Martinez et al., 2006). In addition, a simulation study by Sae‐Lim et al. (2010) showed that different family sizes largely result in unbiased genetic correlation estimates (when *h* ^2^ is moderate and G × E is present) and neither impact their SEs when the REML estimation method is used. It can thus be concluded that a more heritable trait requires a lower sample size per family than a less heritable trait. Hence, the variance estimates and their SEs can presumably be considered reliable in our data structure, where the family number of sampled individuals was less than seven only in seven families in the seminatural environment.

### 4.3 Conservation and evolutionary implications

The Lake Saimaa salmon has not completed its natural life cycle for nearly six decades due to lack of suitable river habitats for spawning and parr/smolt production. This, combined with repeated severe genetic bottlenecks and a long residence time in captive breeding, makes the population of great conservation concern and restoration attempts of a self‐sustaining wild stock a very challenging task. In the present study, both maternal and genetic effects proved to be an influential component of early life fitness variation within the landlocked salmon population, directing selection according to the environmental conditions experienced. A constant amount of additive genetic variation and high heritability estimates in juvenile growth across the tested environments suggest that the study population still has evolutionary potential—irrespective of its very small effective size and low overall diversity—to adaptively respond to an environmental shift from captive rearing back to the wild. This may be interpreted as good news for the future conservation and re‐establishment programs of this critically endangered salmonid.

We additionally conclude that long‐term preservation in captivity has maintained heritable variation within the growth traits of the study population, rather than favoring an optimal phenotype. Nevertheless, the seminatural environment appears to select partially different fish genotypes than does the standard hatchery rearing environment. Considering that growth traits of juvenile salmonids are genetically and phenotypically associated with age (and tendency) of smolting and sexual maturation (Hecht et al., 2015; Kause et al., 2005), hatchery selection also has the power to direct key components of life history trajectories. Consequently, our work supports the previous view that direct genetic effects can be responsible for the rapid phenotypic divergence of hatchery‐reared and wild salmon (Araki et al., 2007, 2009). To mitigate unintended genetic and/or phenotypic changes of captive rearing that adversely influence population restoration efforts, exposure of salmon juveniles (brood fish candidates) to natural‐like conditions during early rearing could be employed as an advanced management means. Such rearing techniques could retain components of natural selection for the critical life stages and thus favor gene combinations better for thriving in the wild.

## FUNDING INFORMATION

Funding was provided by Natural Resources Institute Finland (Luke: ViableSalmon project), Raija and Ossi Tuuliainen Foundation (project 641158), Ministry of Agriculture and Forestry (VN/3589/2023), and University of Eastern Finland.

## CONFLICT OF INTEREST STATEMENT

The authors declare no conflict of interest exists.

## Supporting information


Figure S1.

## Data Availability

Data for this study are available at Mendeley Data repository https://doi.org/10.17632/s33xxrcttz.1.
