# Supplementary material for: Selection among critically endangered landlocked salmon (Salmo salar m. sebago) families in survival and growth traits across early life stages and in different environments
Source: Evol Appl. 2024 Apr 26;17(4):e13692. doi: 10.1111/eva.13692 (PMC11052761; doi:10.1111/eva.13692)
Supplement: Supplementary file 1 — Figure S1. [file EVA-17-e13692-s001.docx]

**APPENDIX 1**


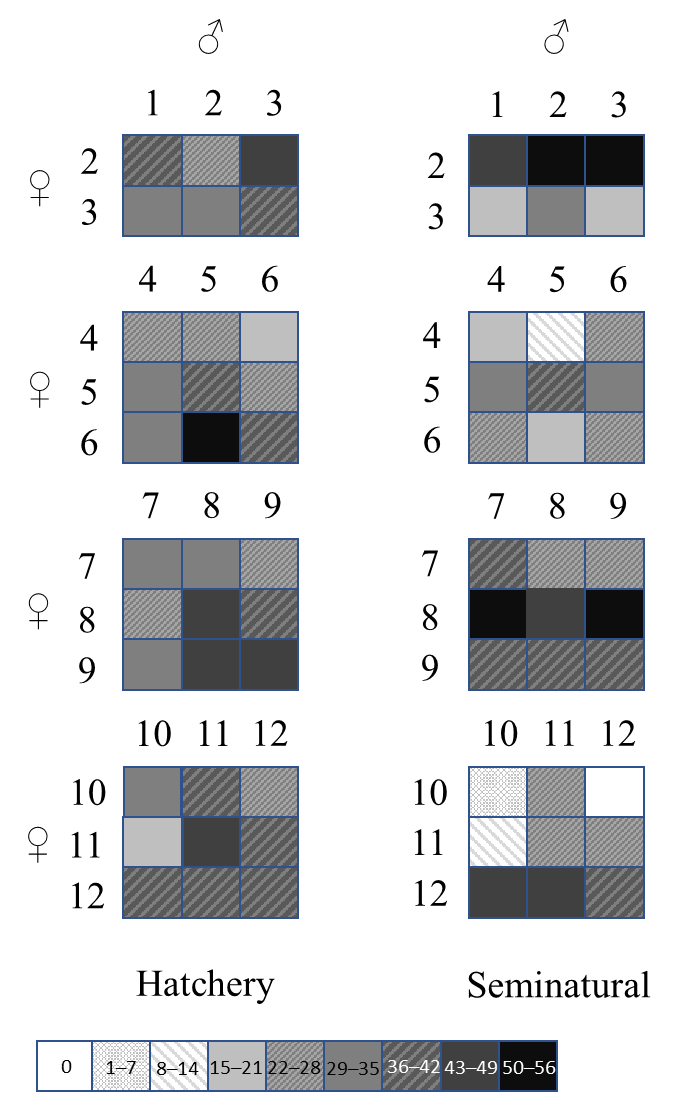


***Fig. S1.*** *Percentage classes of sampled one-summer-old landlocked salmon within families, relative to their total initial number (at swim-up alevin stage) in two rearing environments (hatchery vs. semi-natural). In two families, the initial number of fish was 19+19 (♀6 × ♂5) and 30+29 (♀7 × ♂9) in the hatchery + semi-natural environments. For all other families, the initial number of fish was 42+35, respectively.*
